# Independent effects of 2-D and 3-D locations of stimuli in a 3-D display on response speed in a Simon task

**DOI:** 10.3389/fpsyg.2015.01302

**Published:** 2015-09-01

**Authors:** Hiroyuki Umemura

**Affiliations:** Medical and Biological Engineering Research Group, Biomedical Research Institute, National Institute of Advanced Industrial Science and TechnologyIkeda, Japan

**Keywords:** Simon effect, 3-D, stimulus–response compatibility, reaction time, binocular disparity

## Abstract

The Simon Effect is a phenomenon in which reaction times are usually faster when the stimulus location and the response correspond, even if the stimulus location is irrelevant to the task. Recent studies have demonstrated the Simon effect in a three-dimensional (3-D) display. The present study examined whether two-dimensional (2-D) and 3-D locations simultaneously affected the Simon effect for stimuli in which a target and fixation were located on the same plane (ground or ceiling) at different 3-D depths, and the perspective effect produced a difference in the 2-D vertical location of the target stimulus relative to the fixation. The presence of the ground and ceiling plane was controlled to examine the contextual effects of background. The results showed that the 2-D vertical location and 3-D depth simultaneously affected the speed of responses, and they did not interact. The presence of the background did not affect the magnitude of either the 2-D or the 3-D Simon effect. These results suggest that 2-D vertical location and 3-D depth are coded simultaneously and independently, and both affect response selection in which 2-D and 3-D representations overlap.

## Introduction

When people perform a choice reaction time (RT) task, the time needed to make a response varies with the compatibility between the stimulus and the response. In the spatial case of this stimulus–response (S–R) compatibility effect, RTs are usually faster and responses are more accurate when the stimulus occurs in the same relative location as the response. The Simon Effect is a specific case of the S–R compatibility effect in which the stimulus location is irrelevant to the task (Simon and Rudell, 1967). For example, participants are instructed to press a right key whenever they observe a red target and a left key in response to a white target. Even though stimulus location is entirely task-irrelevant, responses are typically faster when response keys spatially correspond to the stimulus location: red on the right or white on the left. The Simon effect has been extensively investigated, not only because the effects are useful in the design of man–machine interfaces, but also because they provide important insights on attentional operations, the representation of space and body, the cognitive representation of intentional action, and decision making and action execution (Kornblum et al., 1990; Hommel, 2011).

Most studies on the Simon effect have been conducted with two-dimensional (2-D) stimulus displays. Recently, Rigon et al. (2011) showed that the Simon effect is not confined to 2-D displays but can be observed for stimulus locations in depth in three-dimensional (3-D) space defined by binocular disparity (provided by a color anaglyph system or a VR headset). Participants responded faster when the stimulus location (near or far) and the location of response keys were identical. Stins and Michaels (2010) investigated the effect of 3-D background on the S–R compatibility effect. They used a joystick to collect responses and found that RT was affected by 3-D orientation of the background, as defined by a texture gradient (i.e., monocular depth cue). They found an S–R compatibility effect between the 3-dimensional ‘far’ location and the response made by moving a joystick toward ‘far’ (but the effect was not observed when the target was displayed at a near distance in their study). In these and other studies on the S–R compatibility effect in 3-D locations (Chan and Chan, 2010), the effects of 2-D location were regarded as a disturbance. Therefore, 2-D locations were counterbalanced and were not analyzed. As a result, it is not known whether 2-D and 3-D locations simultaneously affect response speed.

It is important to examine whether the 3-D and 2-D locations simultaneously influence the spatial correspondence effect, because this can help explain how the representations of target location and response are coded. In the formulation of the Simon effect, it is assumed that a spatial code is generated for the stimulus location which is irrelevant to the task, and that the Simon effect occurs at the response selection stage (Stoffer and Umiltà, 1997). A stimulus automatically activates the response code that spatially corresponds to it if there is sufficient similarity between the spatial stimulus dimension and the spatial response dimension (Kornblum et al., 1990). When the activated response code is different from the response code required by the task, a conflict is generated that requires time to resolve.

**FIGURE 1:**
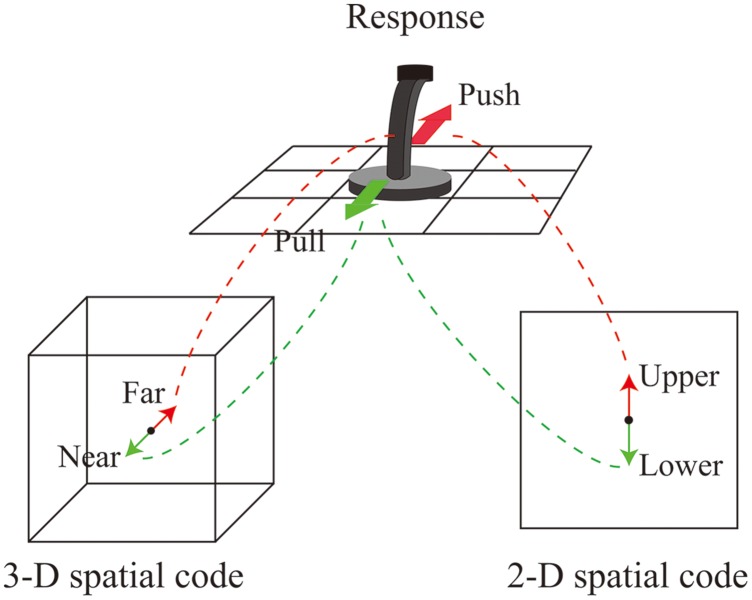
**Illustration of the relationship among two-dimensional (2-D) and three-dimensional (3-D) response codes and the action with the response device**.

The present study examined how 2-D (vertical) location and 3-D location are coded and how these representations interact. One hypothesis is that both the 2-D and 3-D locations of the stimulus influence the speed of responses. Previous research has shown that multiple spatial codes influence the speed of a stimulus identification task. Lamberts et al. (1992) compared the spatial S–R compatibility among eight different positions which were obtained as a result of orthogonal manipulation of hemispace, visual hemifield within hemispace, and relative position within hemifield. They found compatibility effects based on both hemifield and relative position. Hommel and Lippa (1995) showed that object-based spatial stimulus codes are formed automatically and thus influence the speed of response selection. They had participants respond to a stimulus superimposed on the eyes of an image of a face. They found that when the response location was identical to the position of the eye (e.g., ‘left’ response for stimuli on the left eye), the response was relatively fast even if the stimulus was aligned vertically by tilting the face 90 or 270°. This means that the tilted image of face provided the object based spatial relationship. Proctor et al. (2003) used a display in which a target stimulus appeared in one of four corners and response keys were placed diagonally (top-right vs. bottom-left or top-left vs. bottom-right); they obtained a Simon effect for both horizontal and vertical dimensions concurrently. Based on these studies, it is predicted that both the 2-D location and the 3-D location should form different spatial stimulus codes, and both should produce the Simon effect. The situation of the present experiment was similar to the study of Proctor et al. (2003). A joystick ordinarily used to navigate a plane in a 3-D flight simulator was chosen as the response device. The response made with the device implicates not only a 3-D spatial code but also a 2-D spatial code. Because most interfaces assigned to the control of a 2-D display use an arrangement in which inclining the joystick (or pushing a button) away from the user corresponds to the upper 2-D direction, the action of inclination to indicate “far” also contains the response code “upper” in the 2-D vertical location (**Figure 1**).

**FIGURE 2:**
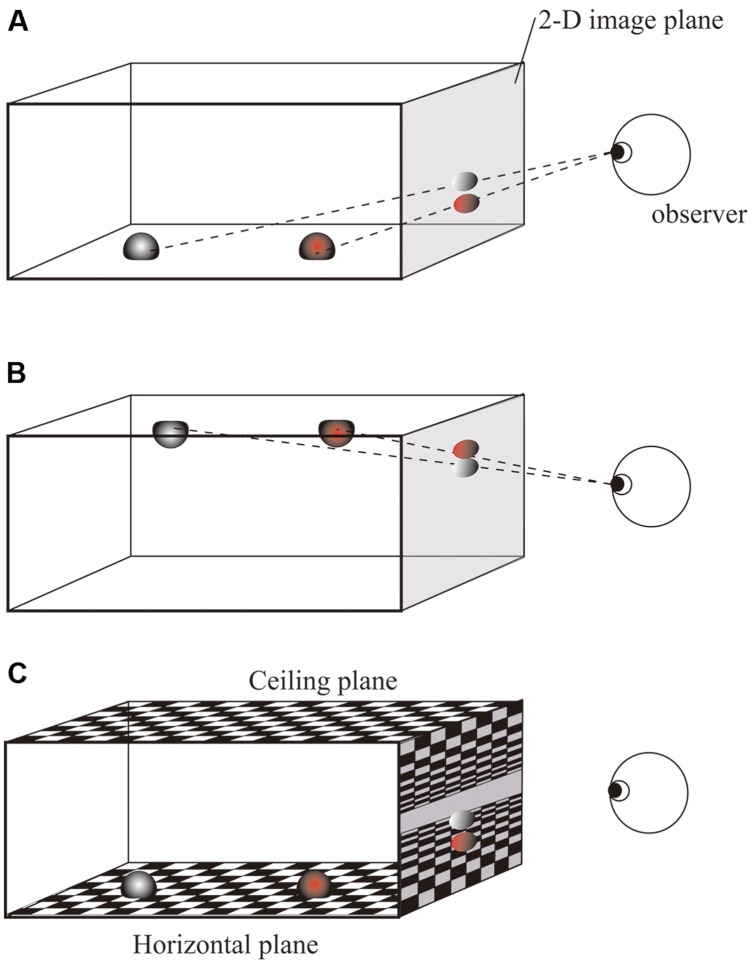
**Illustration of the relationship between 2-D vertical position and 3-D depth caused by the perspective effect. (A)** Two objects have different heights on the 2-D image due to their difference in 3-D horizontal distance (depth). **(B)** When two objects are placed on the ceiling, the farther object is lower on the 2-D image. **(C)** Textured ground gives a strong impression that the two objects are at the same height in 3-D space.

An alternative possibility is that the 2-D location has no or little effect on the speed of response. This hypothesis seems unlikely because the 2-D Simon effect has been repeatedly reported. Consider, however, the display in **Figures 2A,B**, in which a red object and a gray object are located at the same vertical height (i.e., on the same horizontal plane) in a 3-D scene. Because they differ in depth relative to the observer, they appear to have different vertical locations on the 2-D image due to projection. The present study focused on this 2-D vertical difference produced by projection from a 3-D scene onto a 2-D image. In such a case, it might be possible that the 2-D Simon effects are decreased, or in the extreme case, disappear, if the locations objects are coded in 3-D representation when they are embedded in 3-D space. Furthermore, the relative impact of 2-D and 3-D representations on the Simon effect may be modified by additional information about the context in which the stimuli are embedded in the 3-D environment. The present study therefore included a condition with a background which is composed of textured ground and ceiling planes. (**Figure 2C**). These planes are parallel to the horizontal plane, and positing a fixation and target objects on the same plane should strengthen a context in which the two objects are at the same vertical positions in 3-D space. If the existence of the background provides contextual information, as reported in previous studies (Hommel and Lippa, 1995; Stins and Michaels, 2010), the relative effects of 2-D and 3-D information should be altered. To examine this, ground and ceiling planes were present in Experiment 1 and absent in Experiment 2. In both experiments, 2-D vertical positions of the targets were produced by effects of perspective. When the background was absent, the impression that the fixation and target stimuli were located at the same height in the 3-D scene was reduced.

## Materials and Methods

### Participants

Eighteen people (12 males and 6 females) participated in both Experiments 1 and 2. All were between 20 and 30 years of age and had normal or corrected-to-normal vision. None were aware of the purpose of the present experiment. Written informed consent was obtained from all participants before the experiment. All the experimental procedures were approved by the Ethics Committee for Human and Animal Research of the National Institute of Advanced Industrial Science and Technology (AIST).

### Apparatus and Setups

Experiments were conducted in a dark room. A Windows PC was used to control stimulus presentation on a CRT monitor (Sony 24^′′^ GDMFW900) placed at 75 cm distance from the observer. The height of the center of the display and the eye-height of each participant was adjusted by a chin-rest. To display the stimuli with binocular disparity, a shutter goggle (Stereographics, Crystal eyes) was used. The CRT’s refresh rate was 85 Hz and its resolution was 1024 × 768. A joystick, Thrustmaster T-Flight stick X, was used for making responses. This joystick had a conventional design of a stick for aircraft, with a stem about 18 cm in height and 4 cm in diameter. The joystick was placed in front of the participant and the two response directions (near–far) were along their midline. Participants grasped the joystick stem with their dominant hand and held its base down with their non-dominant hand.

### Stimuli and Tasks

**FIGURE 3:**
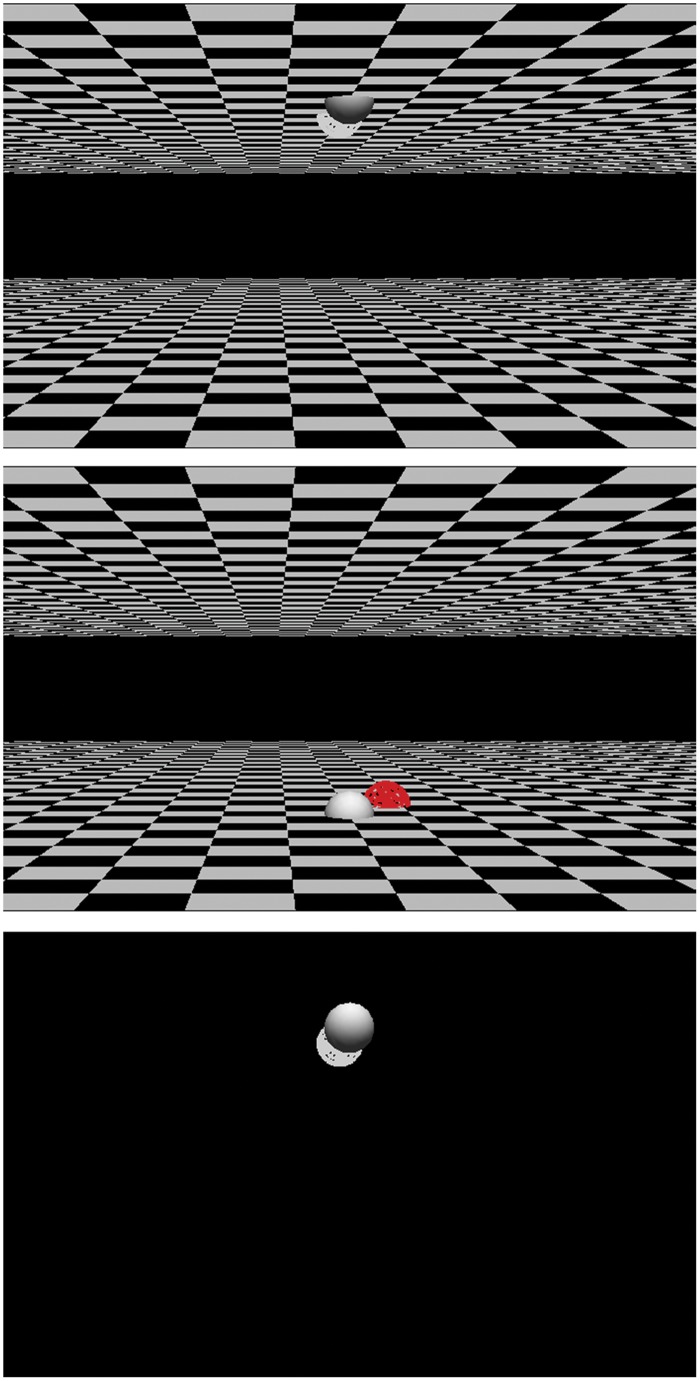
**Examples of stimuli.** Solid spheres are fixation and wired spheres are target stimuli. **(top,middle)**: Stimuli in Experiment 1. **(bottom)**: Stimulus in Experiment 2. The location of the target in the **(bottom)** is the same as that in the **(top)** in the experiment They were 3-dimensionally provided through a stereo shutter glass.

In both Experiments 1 and 2, participants were required to respond to the color of the target stimulus. The target stimulus was presented at far or near depths relative to a fixation stimulus. The 2-D vertical location of the target stimulus (upper or lower) was produced according to the perspective effect (**Figure 3**). The location of the stimulus and the color of the stimulus were independently determined, and participants were required to ignore the location of the stimulus.

The positions of the fixation point and a target stimulus were determined in a 3-D space. The following procedure was used to draw a stimulus display in Experiment 1, in which the background (ground plane and ceiling plane) was present. Except for the presence of the background, the same procedure was used in Experiment 2. A ground plane and a ceiling plane were drawn 10 cm (in the 3-D scene) below and above eye height. They extended 50 cm away from the fixation and the observer (*far side* of the fixation) and 15 cm from the fixation toward the observer (*near side*). On the 2-D image, the planes created rectangles at a height of 5° of visual angle, with a 3° gap at the center of the image (**Figure 3**). These planes were textured with a checkerboard, and the gap was black. The fixation and target stimulus were placed on the same plane. The fixation was a solid sphere with a radius of 1 cm in 3-D space (0.8° of visual angle) and was placed on the ground or ceiling plane. It was located 75 cm away from the observer in 3-D and 7.6° below or above the center of the CRT. The target stimulus was a wire-framed sphere colored red or white. Its size was as same as that of the fixation stimulus in 3-D space, but this varied on the 2-D display according to perspective. The target stimulus was located on the near side or far side of the fixation. The 2-D vertical position of the target was determined by the combination of the depth of the target and the plane on which the target and the fixation were located. For example, when the fixation and the target stimulus were placed on the ceiling plane, the target stimulus on the far side appeared below the fixation on the 2-D image (**Figure 3**, top). In this case, if the correct response was pulling the joystick toward the observer (near), the 2-D vertical location was compatible but the 3-D depth was incompatible. The relationships among the depth/plane combinations and response-location compatibilities are summarized in **Table 1**. The distance of the target stimuli from the fixation in 3-D space varied from 4 to 8 cm far or near. As a result, the vertical distance between the target stimuli and fixation stimuli on 2-D display varied from 0.5 to 1.4° when the target was positioned on the near side in 3-D, and 0.4 to 1.0° at the upper when the target was positioned on the far side in 3-D. Horizontal distance also varied from -1.1 to 1.1° (2.5 cm in 3-D), but this was not analyzed.

**Table 1:** The relationships among response-location compatibilities and combinations of the depth of the target and the plane on which the target and the fixation were located.

Plane	Ground				Ceiling			
Target position relative to fixation	Far		Near		Far		Near	
Response direction	Far	Near	Far	Near	Far	Near	Far	Near
2-D location-response compatibility	+	-	-	+	-	+	+	-
3-D location-response compatibility	+	-	-	+	+	-	-	+

*The symbols ‘+’ indicates compatibility between the stimulus location and response and ‘-’ indicates incompatibility.*

On each trial, the fixation was displayed on the ground or ceiling plane (these planes were invisible in Experiment 2). The participants were required to gaze at the fixation and initiated a trial by pressing a button on the joystick. After 1.5 s, a target stimulus was displayed. Participants responded to its color by pushing (inclining toward the far side) or pulling (inclining toward the near side) the joystick. The correspondence between the direction and the color was counterbalanced among participants; that is, half of the participants responded to the red stimulus by pushing the joystick and responded to the white stimulus by pulling. Participants were required to respond as fast and as accurately as possible.

In Experiments 1 and 2, participants were presented with each combination of two target depths (near or far) and two planes (ground or ceiling) 32 times, for a total of 128 trials. A rest break was provided after half of the trials were completed. All participants conducted both experiments; half participated in Experiment 1 first.

## Results

Mean RTs and percent errors (PEs) are summarized in **Table 2**. RTs were categorized by three factors: the conflict between response direction and 2-D vertical location, the conflict between response and 3-D depth, and response direction (see **Table 1**). Here, the 2-D vertical location ‘upper’ was considered to be consistent with the response action of ‘push,’ and ‘lower’ with ‘pull.’ RTs for incorrect responses and for trials on which they were shorter than 150 ms or longer than 2000 ms were excluded. Repeated-measure ANOVAs with three within-subjects factors (two 3-D depths, two 2-D vertical locations, and two response directions) were conducted on RTs and PEs.

**Table 2:** Mean RTs, (in milliseconds), standard deviations (*SDs*), differences (ΔRT = RT for incompatible – RT for compatible), and percent errors (PEs) in Experiments 1 and 2 as a function of 3-D depth-response correspondence, 2-D vertical location-response correspondence, and correct response action.

	RT (*SD*)			PE	
	3-D location-response compatibility			3-D location-response compatibility	
2-D location-response compatibility	+	-	ΔRT(3D)	+	-
**Experiment 1 (background was present)**
	Correct response: Near			
+	459 (51)	496 (67)	37	3.1	4.9
-	474 (58)	507 (53)	33	0.7	7.1
ΔRT(2D)	15	11		
	Correct response: Far			
+	464 (48)	494 (49)	30	1.6	5.4
-	484 (50)	509 (63)	25	4	6.7
ΔRT(2D)	20	15			

**Experiment 2 (background was absent)**	
	Correct response: Near			
+	462 (54)	481 (67)	19	3.6	6.1
-	477 (56)	515 (84)	38	4.7	4.2
ΔRT(2D)	15	34			
	Correct response: Far			
+	468 (56)	493 (58)	25	1.1	4
-	483 (70)	510 (73)	27	4.1	6.1
ΔRT(2D)	15	17			

*The symbol ‘+’ indicates compatibility between the stimulus location and response and ‘-’ indicates incompatibility.*

ANOVA for the RTs in Experiment 1, in which the ground and ceiling planes were displayed (**Table 2**, top), revealed significant main effects of 3-D depth [*F*(1,17) = 25.456, *p* < 0.001, $\eta_{p}^{2}$ = 0.6] and 2-D vertical location [*F*(1,17) = 10.491, *p* < 0.005, $\eta_{p}^{2}$ = 0.382], but not response direction [*F*(1,17) = 0.634, *p* = 0.437, $\eta_{p}^{2}$ = 0.036]. All interactions between and among these factors were not significant. ANOVA for the PEs revealed significant main effects of 3-D depth [*F*(1,17) = 4.776, *p* = 0.043, $\eta_{p}^{2}$ = 0.219] but not 2-D vertical location [*F*(1,17) = 0.676, *p* = 0.422, $\eta_{p}^{2}$ = 0.038], and response direction [*F*(1,18) = 0.295, *p* = 0.594, $\eta_{p}^{2}$ = 0.017]. Although significant main effects were observed only for effects of 3-D depth, the pattern of effects for PEs and RTs did not conflict.

In Experiment 2, in which the ground and ceiling planes were invisible (**Table 2**, bottom), ANOVA for the RTs revealed significant main effects of 3-D depth [*F*(1,17) = 15.201, *p* = 0.001, $\eta_{p}^{2}$ = 0.472] and 2-D vertical location [*F*(1,17) = 18.523, *p* < 0.001, $\eta_{p}^{2}$ = 0.521], but not response direction [*F*(1,17) = 0.826, *p* = 0.376, $\eta_{p}^{2}$ = 0.046]. All interactions of these factors were not significant. ANOVA for the PEs revealed no significant main effects of 3-D depth [*F*(1,17) = 2.181, *p* = 0.158, $\eta_{p}^{2}$ = 0.114], 2-D vertical position [*F*(1,17) = 1.527, *p* = 0.233, $\eta_{p}^{2}$ = 0.082], or response direction [*F*(1,17) = 0.595, *p* = 0.451, $\eta_{p}^{2}$ = 0.07]. Although no significant main effects were observed, the pattern of effects for PEs and RTs did not conflict.

**FIGURE 4:**
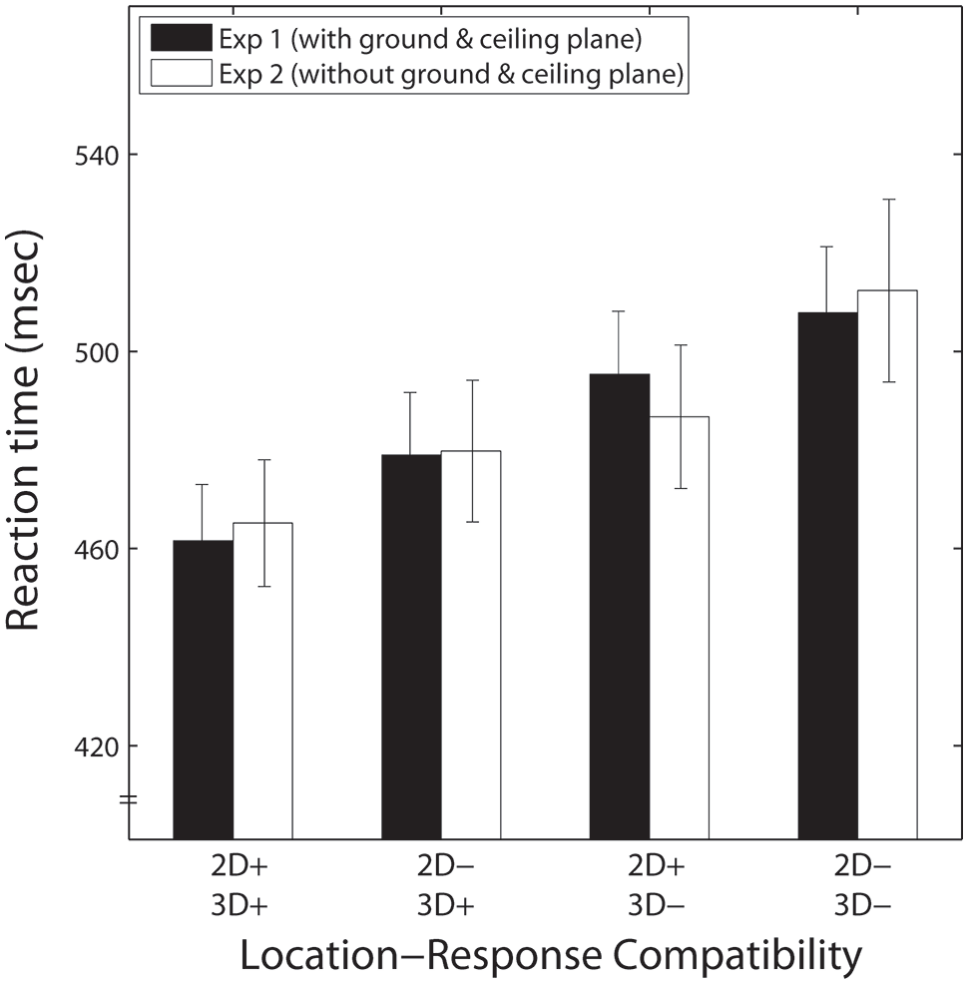
**Mean reaction times (RTs) for Experiment 1 (black bars) and Experiment 2 (white bars) as a function of location-response compatibility.** Error bars indicate *SEs*. The symbol ‘+’ indicates compatibility between the stimulus location and response, and ‘-’ means incompatibility.

To examine the effect of the presence of the background, a repeated measures ANOVA with three within-subjects factors (presence of background, 3-D depth, and 2-D vertical location) was conducted on the RTs merged across the two response directions in each of the experiments; response directions were non-significant in the previous ANOVAs. These merged RTs are shown in **Figure 4**. The results of the ANOVA showed no significant effect of the presence of the background [*F*(1,17) = 0.001, *p* > 0.5]. The main effects of 2-D vertical location and 3-D depth were significant [*F*(1,17) = 29.802, *p* < 0.001, *F*(1,17) = 26.236, *p* < 0.001], and no significant interactions were observed for 2-D vertical location and 3-D depth [*F*(1,17) = 0.302, *p* > 0.5], presence of the background and 2-D vertical location [*F*(1,17) = 0.676, *p* = 0.422], and presence of the background and 3-D depth [*F*(1,17) = 0.381, *p* > 0.5]. This indicates that the presence of the background did not affect the 2-D and 3-D Simon effects.

## Discussion

Experiments 1 and 2 both clearly showed that 3-D depth and 2-D vertical locations of the target affected the speed of responses. The lack of interaction between 3-D depth and 2-D vertical location indicated that the incompatibility of 2-D vertical location and response direction and those in 3-D depth had additive effects on the Simon effect. This suggests that the codes of the 2-D vertical location and the 3-D depth were independently and simultaneously formed, and these independently prolonged the response if each or both of the location codes conflicted with the response made by the joystick, in which 2-D vertical location and 3-D depth overlapped. Previous research has suggested that the human brain codes several spatial aspects (Lamberts et al., 1992; Hommel and Lippa, 1995; Proctor et al., 2003; Valle-Inclán et al., 2003; Rubichi et al., 2006). 3-D depth should form another such spatial dimension, and a stimulus in 3-D space should be defined by a combination of these codes.

Generally, Experiments 1 and 2 produced similar patterns of response. One of the predicted outcomes was that the 2-D vertical locations would be less effective when the ceiling and ground planes were simultaneously displayed. That is, the existence of the ground and ceiling planes should decrease the Simon effect for the 2-D vertical locations, because these planes provide the context that the fixation and the target lay on the same plane. This was not observed. The presence of the background had no influence on the speed of response. The absence of these effects suggests that 2-D information is coded based on the retinal image rather than reconstructed 3-D scene. These results are interesting because previous research showed that contextually given location affected the speed of responses (Hommel and Lippa, 1995). This difference may have resulted because background played different roles in these studies. The context given by the background used in Hommel and Lippa (1995) (a picture of tilted face) contained a different (new) spatial frame, and this was superimposed on the original one (i.e., body-centered); spatial representations are coded based on both the new context and original frames. On the other hand, the context given by the background in the present study did not add a new frame. The context might weakened the relative impression of 2-dimensionality, but the compatibility effect, once triggered with the retinal image, could not be canceled by the contextual information acquired during the 3-D reconstruction processing that follows.

In both experiments, the effects of depth were larger than those of 2-D vertical location. The effect of 3-D depth, or the mean RT difference for trials in which 3-D depth and response direction conflicted versus those with no conflict [mean ΔRT(3D) in **Table 2**] was 31 ms in Experiment 1 and 27 ms in Experiment 2. On the other hand, the effect of 2-D location [mean ΔRT(2D) in **Table 2**] was 15 ms in Experiment 1 and 20 ms in Experiment 2. Although the size of the Simon effect was larger for 3-D depth than for 2-D vertical location, the relative strength between 2-D and 3-D locations cannot be determined, because the present experiment did not match the distances of these two directions. Shifts in 2-D vertical position were very small because they were produced by changes in relative height due to perspective. Proctor et al. (2003) examined whether the relative magnitudes of the horizontal and vertical Simon effects could be systematically altered by manipulating the relative distance of the horizontal and vertical dimensions. They showed that the magnitude of the vertical Simon effect changes with the manipulation of the distance. This means that further experiments are required to determine the relative strengths of the 2-D vertical Simon effect and the 3-D depth Simon effect. What is important in the present results is that the 2-D Simon effect was observed even with a small shift in 2-D vertical location.

In the present study, there was no significant difference between responses corresponding to ‘near’ and ‘far’ locations. Stins and Michaels (2010), who investigated the effect of the presence of a 2-D textured background on the S–R compatibility effect, reported an asymmetrical effect of depth direction. They found an S–R compatibility effect between the 3-dimensional ‘far’ location and the response made by moving a joystick away (toward ‘far’), but the effect was not observed when the target was displayed at a near distance. This difference probably arose from the richness of 3-D depth information in the display. Unlike the display used by Stins and Michaels (2010), the display in the present experiment involved binocular disparity, relative size, and occlusion when necessary. The display of Stins and Michaels (2010) could not provide sufficient depth information in near space because the texture was sparse there. The richness of the 3-D information in the present display may also have contributed to the absence of a significant effect of the presence of the background. Even if the presence of background had no contextual effect, it could have been used as a cue for 3-D depth. However, it seems that binocular disparity and relative size provided sufficient 3-D information in the present experiment.

The main finding of the present study is that conflicts in 3-D depth and 2-D vertical location independently and simultaneously affected performance. The results are consistent with previous studies which showed the effect of multiple frames. With respect to the formation of spatial coding, there is a debate between the referential coding account (Umiltà and Nicoletti, 1985; Hommel, 1993, 2011) and the attentional shift account (Nicoletti and Umiltà, 1994; Proctor and Lu, 1994; Stoffer and Umiltà, 1997) about when and how the code is formed. Although the present experiments did not intend to reveal which of these accounts is preferable, the results are suggestive. The referential coding account (Umiltà and Nicoletti, 1985; Hommel, 1993, 2011) assumes that relative spatial coding is accomplished by relating the target stimulus to reference frames or reference objects. The attentional shift account holds that a spatial code is generated through a shift of attention to the location of the target stimulus (Nicoletti and Umiltà, 1994; Stoffer and Umiltà, 1997). One important difference between the two accounts is that the attentional shift account cannot explain a Simon effect that occurs in multiple frames, because it assumes that there can be only one relative spatial code that is automatically generated for one attentional shift (Stoffer and Umiltà, 1997). Therefore, the attentional shift account seems to require some modification to provide an account for the present results. While, the referential-coding account can readily provide an account for the present results, because the account allows parallel coding of a stimulus if multiple frames of references are available.

It has been suggested that the difficulty of accounting for more than one active spatial code (e.g., spatial codes for horizontal and vertical locations) can be resolved by assuming that there are as many attention shifts as relative spatial codes (Stoffer and Umiltà, 1997). This seems applicable to the present results because the movement of attention in depth is known to occur (de Gonzaga Gawryszewski et al., 1987). However, the attentional shift account is based on the premotor theory of attention, in which programming of a saccadic eye movement toward a position is assumed to be necessary in order to shift attention (Rizzolatti et al., 1987, Umiltà et al., 1991, Stoffer and Umiltà, 1997, Van der Lubbe and Abrahamse, 2011). When attention shifts toward a stimulus, the program for the saccadic eye movement is prepared, and this oculomotor program for the saccade becomes a spatial code of the stimulus. Rigon et al. (2011) argued that the existence of the 3-D Simon effect does not support this account, because the saccadic eye movement should occur in the horizontal and vertical dimensions, but not in depth; therefore programming of vergence eye movements are necessary to account for the 3-D Simon effect. The present results also support this view. Reconciliation of the attentional shift account with the 3-D Simon effect may be based on programming of eye movements, including both saccadic and vergence eye movements. Saccadic eye movements should generate 2-D horizontal and vertical spatial codes, and vergence eye movements should generate 3-D depth codes. Yet it is unlikely that only one spatial code can account for the Simon effect. Thus, it seems valid to assume the simultaneous and parallel existence of 2-D and 3-D representations.

The present study reported that 2-D vertical location and 3-D depth are coded simultaneously and independently, and both of them affect response selection, in which 2-D and 3-D representations overlap. The present study used a CRT display with stereo shutter glasses, because these were suitable to control the experiment. The present results, however, should be confirmed in additional experiments in a real environment or in one using virtual reality techniques in which participants can move their heads.

## Conflict of Interest Statement

The author declares that the research was conducted in the absence of any commercial or financial relationships that could be construed as a potential conflict of interest.
